# High Magnitude of Social Anxiety Disorder in School Adolescents

**DOI:** 10.1155/2017/5643136

**Published:** 2017-02-16

**Authors:** Kindie Mekuria, Haregwoin Mulat, Habtamu Derajew, Tesfa Mekonen, Wubalem Fekadu, Amsalu Belete, Solomon Yimer, Getasew Legas, Melak Menberu, Asmamaw Getnet, Simegnew Kibret

**Affiliations:** ^1^Woldia Hospital, Woldia, Ethiopia; ^2^Psychiatry Department, University of Gondar, Gondar, Ethiopia; ^3^Amanuel Mental Specialized Hospital, Addis Ababa, Ethiopia; ^4^Psychiatry Department, Bahir Dar University, Bahir Dar, Ethiopia; ^5^College of Medicine and Health Sciences, Debre Tabor University, Debre Tabor, Ethiopia; ^6^Psychiatry Department, Dilla University, Dilla, Ethiopia; ^7^College of Health Sciences, Mizan-Tepi University, Mizan-Tepi, Ethiopia; ^8^Finote Selam Hospital, Finote Selam, Ethiopia

## Abstract

*Introduction.* Social phobia is the most prevalent and chronic type of anxiety disorder worldwide and it affects occupational, educational, and social affairs of the individual. Social phobia is also known for its association with depression and substance use disorder.* Objective.* The aim of this study was to assess the prevalence and associated factors of social phobia among high school students in Ethiopia.* Methods.* Cross-sectional study was conducted among 386 randomly selected students. Data were collected using pretested and self-administered questionnaire. Social phobia was assessed by using Social Phobia Inventory (SPIN). Logistic regression was used to analyze the data with 95% confidence interval and variables with *p* value less than 0.05 were considered as statistically significant.* Results.* From 386 study participants, 106 (27.5%) of them were positive for social phobia. Being female (AOR = 3.1; 95% CI: 1.82–5.27), current alcohol drinking (AOR = 1.75; 95% CI: 1.03–2.98), poor social support (AOR = 2.40; 95% CI: 1.17–4.92), and living with single parent (AOR = 5.72; 95% CI: 2.98–10.99) were significantly associated with social phobia.* Conclusion.* The proportion of social phobia was higher compared to previous evidences. School-based youth-friendly mental health services might be helpful to tackle this problem.

## 1. Introduction

DSM-V defines social phobia as marked or intense fear or anxiety of social situations in which the individual may be scrutinized by others and this situation interferes significantly with routines, occupational (academic) functioning, social activities, and relationships [1]. Though it is a debilitating psychiatric condition which is treatable, it often remains undetected and untreated. Individuals will have reduced quality of life, disturbed social interactions, poor daily functioning, and poor treatment adherence for other medical or psychiatric conditions [2–4].

The prevalence of social phobia among school adolescents varied from country to country [5]. For instance, in high-income countries, the magnitude ranges from 3.5% to 21% [6–10]. Even though there is scarcity of evidence in developing countries, the available literatures suggested that social phobia is higher, which ranges from 10.3% to 27% [11, 12].

Students with social phobia have difficulty of speaking in front of a group of people and fail or drop out of school due to fear [13]. Students' attention to academic information may be distracted by an excessive focus on their anxiety [14]. The ability to monitor and modify communication with colleagues and teachers may be vague by fear of negative evaluation and when participating in a seminar, socially anxious students judge their competence poorly [15], which leads to academic underachievement [16].

Evidences showed that social phobia was associated with 20% of cases of adult depression [17] and 17% of cases of alcohol and drug dependence [18]. Low socioeconomic status, being unmarried, unemployment, low level of education, and poor social support were identified as a risk factor for social phobia [19]. Society's attitude towards shyness and avoidance as a measure of politeness is also an important factor associated with students' ability to build social interaction [20].

Despite the high worldwide burden of social phobia, limited evidence is available, particularly in developing countries. This study tried to assess factors (social support and wealth index) which were previously not well addressed. The purpose of this study was to assess the prevalence and associated factors of social phobia among school adolescents in Ethiopia.

## 2. Methods and Materials

School-based cross-sectional study was conducted at Woldia preparatory school. A total of 1516 regular students in 11th and 12th grades in 2015 academic year were enrolled. The school is located in Woldia town, Amhara regional state, 521 kilometers away from Addis Ababa. There is one general hospital and two health centers in the town. All students who were enrolled at Woldia preparatory school in 2014/2015 academic year were included in the study. The sample size for this study was calculated by using single population proportion formula and 424 students were recruited randomly.

Social phobia was the dependent variable and the predictors were sociodemographic variables (sex, age, religion, wealth index, relationship status, current living condition, and origin of residence), clinical factors (past history of psychiatric illness, past history of chronic medical illness, and family history of mental illness), substance use (cigarette, Khat chewing, and alcohol use), and social support. Khat (also called Catha edulis) is a local green leaf plant which had amphetamine-like stimulating effect after chewing due to the active ingredient called cathinone.

Data was collected by pretested self-administered questionnaire and the data collection was facilitated by trained data collectors and supervisors. Social phobia was assessed by using the Social Phobia Inventory (SPIN) which is a 17-item self-rating scale developed by Connor and his colleagues [21]. It shows the symptom domains of social phobia (fear, avoidance, and physiological arousal) and appears to have reliable and valid psychometric properties of screening social phobia in adolescents and other populations [21–24]. It was rated from 0 (not at all) to 4 (extremely) and the sum score ranged from 0 to 68 [21]. A student with a score of 20 and above on SPIN was considered as having social phobia.

Oslo 3-item social support scale was used to determine the level of social support. The total score ranged from 3 to 14 and was categorized into poor support (3–8 scores), moderate support (9–11 scores), and strong support (12–14 scores) [25]. Students' substance use was explained by verbal report of consuming of any alcohol, cigarette, and Khat in the previous three months. Presence and absence of major illness (medical or psychiatric) were also based on report of the participants.

Data were entered by Epi-Info 3.5.3 analyzed by SPSS-21 for Windows version. Factors association was done by running bivariate and multivariable logistic regression. Strength of the association was presented by odds ratio with 95% confidence interval and *p* value <0.05 was used to identify statistically significant variables.

Ethical clearance was obtained from Institutional Review Board (IRB) of University of Gondar and Amanuel Mental Specialized Hospital. Confidentiality of the participants was maintained by anonymous questionnaire. Participants were fully informed about the purposes of the study prior to the data collection and informed consent (assent) was obtained.

## 3. Results

### 3.1. Sociodemographic Characteristics of Respondents

A total of 424 students were included in the study, of which 386 completed the questionnaires with the response rate of 91.03%. 233 (60.4%) of the participants were males and 346 (89.6%) were between the ages of 16 and 19 years. The mean age of the respondents was 18.15 ± 1.24 years (Table 1).

### 3.2. Clinical and Psychosocial Characteristics of the Respondents

Fourteen (3.6%) students had past psychiatric history and 18 (4.7%) of them had family history of mental illness. About one-third of the participants reported that they did not use any mass media (Table 2).

### 3.3. Prevalence and Associated Factors of Social Phobia

The overall prevalence of social phobia was 27.5%. In multivariable logistic regression analysis, sex, current use of alcohol, social support, and students raised by single parent were significantly associated with social phobia (Table 3).

## 4. Discussion

The overall prevalence of social phobia was 27.5% and it was consistent with the study done elsewhere which was 26.5% [26]. However, the present study was higher than the study conducted in Nigeria, 9.4% [27], Swedish high school students, 21% [9], Iran, 6.2% [6], Delhi, 10.3% [11], and china, 14.1% [12], as well as the national comorbidity survey in USA, 9% [8]. It was also higher compared to a comparative study among high school students in Egypt, Saudi Arabia, and United Arab Emirates where the percentages were 13%, 9.8%, and 7.8%, respectively [28]. This discrepancy might be due to sociocultural differences between those countries and Ethiopia. For instance, in Ethiopia, shyness as a measure of politeness has been emphasized as a dominant cultural norm [23]. The other possible reason for this difference might be the tool used in the current study, which is nondiagnostic self-administered tool, and this might overestimate the prevalence of social phobia among students [21, 22, 24].

In this study, social phobia had significant association with sex, current alcohol drinking, social support, and students' living status. In female students, the odds of having social phobia were 3 times more than those of male students and this was supported by the study done in Poland [26] and Baghdad [29]. The community's perception towards shyness and politeness as a measure of predominant cultural norm might have high influence on female students' social phobia status [23]. Students who were current alcohol users were about 2 times more likely to have social phobia as compared with their counterparts. Students with social phobia tend to engage themselves into heavy drinking and alcohol use problems [30]. This finding was consistent with previous studies conducted in Nigeria [27] and USA [10]. The students probably use alcohol as self-medication for their fear and for their concerns of negative evaluation by others [18].

Social support was another important factor associated with social phobia. Students with poor and moderate social support had about two times more odds to have social phobia as compared with students who had strong social support and the finding was in line with the study done in Iran [7]. Social connectedness is important for the development of confidence and positive social skills. In addition to poor social support, students who lived with single parent were more than 5 times more likely to have social phobia as compared with students who lived or were raised by both parents. This finding was supported by study done in Saudi Arabia [31]. However, students' media usage, wealth index, past psychiatric history, and family history of mental illness did not show any association with social phobia in this study.

## 5. Limitations of the Study

The first limitation of the study may be the fact that we used nondiagnostic self-rating screening tool; the prevalence of social phobia might be overestimated. The other limitation is that we did not measure substance use by a standard tool and consuming a substance for one day and daily use of a substance were reported as substance use, and the association may not be real if standard tool has been used. The last limitation is our design which cannot show the direction of association.

## 6. Conclusion

The proportion of social phobia was higher which was predicted by female gender, current alcohol use, living with single parent, and having poor social support. School and community intervention on students' alcohol use is important. Moreover, school-based youth-friendly mental health service might be very helpful to tackle this problem and to help students with this problem. Further longitudinal and interventional studies are needed to describe the longitudinal nature of the illness and to identify risk factors.

## Figures and Tables

**Table 1:** Sociodemographic distribution of the respondents (*n* = 386).

Sociodemographic variables	Frequency	Percent
Sex		
Male	233	60.4
Female	153	39.6
Age		
16–19	346	89.6
>19	40	10.4
Relationship status		
Single	350	90.4
Married	36	9.6
Ethnicity		
Amhara	377	97.7
Others	9	2.3
Religion		
Orthodox	304	78.8
Muslim	71	18.4
Protestant	11	2.8
Place of upbringing		
Urban	236	61.1
Rural	150	38.9
Lived with		
Both parents	267	69.2
Single parent	61	15.8
Relative/alone	58	15
Semester result (performance)		
Low	236	61.2
Medium	119	30.8
High	31	8.0
Wealth index		
Lowest (poor)	35	9.1
Second	151	39.1
Medium	46	11.9
Fourth	109	28.2
Highest (rich)	45	11.7

*Note*. Ethnicity (others) = Tigre, 6, and Afar, 3; performance (average academic score): low ≤ 70, medium = 70–84.99, and high ≥ 85.

**Table 2:** Clinical conditions of respondents (*n* = 386).

Variables	Frequency (*n*)	Percent (%)
Past psychiatric history		
Yes	14	3.6
No	372	96.4
Family psychiatric history		
Yes	18	4.7
No	368	95.3
Previous medical illness		
Yes	37	9.6
No	349	90.4
Social support		
Poor	65	16.8
Moderate	159	41.2
Strong	162	42.0
Using mass media		
Yes	274	71.0
No	112	29.0
Using social media		
Yes	220	57.0
No	166	43.0
Alcohol		
Yes	135	34.9
No	251	65.1

**Table 3:** Factors associated with social phobia.

Variables	Social phobia		COR (95% CI)	AOR (95% CI)
	Yes	No		
Sex				
Male	41	192	1.00	1.00
Female	65	88	3.44 (2.17, 5.51)	3.10 (1.82,5.27)^**∗**^
Age (years)				
16–19	102	244	3.76 (1.31, 10.84)	2.02 (.66, 6.26)
>19	4	36	1.00	1.00
Alcohol (current use)				
Yes	47	88	1.74 (1.10, 2.75)	1.75 (1.03,2.98)^*∗ ***∗**^
No	59	192	1.00	1.00
Psychiatric history				
Yes	7	7	2.76 (.94, 8.06)	3.15 (.97, 10.29**)**
No	99	273	1.00	1.00
Social support				
Poor	25	40	2.75 (1.45, 5.20)	2.40 (1.17,4.92)^*∗ ***∗**^
Moderate	51	108	2.08 (1.24, 3.49)	2.14 (1.20,3.80)^*∗ ***∗**^
Strong	30	132	1.00	1.00
Living status (students were raised by or lived with)				
Both parents	53	214	1.00	1.00
Single parent	38	23	6.67 (3.67, 12.14)	5.72 (2.98,10.99)^**∗**^
Alone/others	15	43	1.41 (.73, 2.73)	1.86 (.91, 3.82)

Note: ^*∗*^*p* < 0.001 and ^*∗∗*^*p* < 0.05.
